# Imaging tripartite synapses using super-resolution microscopy

**DOI:** 10.1016/j.ymeth.2019.05.024

**Published:** 2020-03-01

**Authors:** Janosch Peter Heller, Tuamoru Odii, Kaiyu Zheng, Dmitri A. Rusakov

**Affiliations:** a UCL Queen Square Institute of Neurology, University College London, London, United Kingdom; b FutureNeuro Research Centre, Royal College of Surgeons in Ireland, Dublin, Ireland; c Department of Physiology, Faculty of Basic Medical Sciences, Alex Ekwueme Federal University Ndufu-Alike Ikwo, PMB 1010 Abakaliki, Nigeria

**Keywords:** Tripartite synapses, Super-resolution microscopy, SMLM, dSTORM, Immunohistochemistry, Astrocytes

## Abstract

Astroglia are vital facilitators of brain development, homeostasis, and metabolic support. In addition, they are also essential to the formation and regulation of synaptic circuits. Due to the extraordinary complex, nanoscopic morphology of astrocytes, the underlying cellular mechanisms have been poorly understood. In particular, fine astrocytic processes that can be found in the vicinity of synapses have been difficult to study using traditional imaging techniques.

Here, we describe a 3D three-colour super-resolution microscopy approach to unravel the nanostructure of tripartite synapses. The method is based on the SMLM technique direct stochastic optical reconstruction microscopy (dSTORM) which uses conventional fluorophore-labelled antibodies. This approach enables reconstructing the nanoscale localisation of individual astrocytic glutamate transporter (GLT-1) molecules surrounding presynaptic (bassoon) and postsynaptic (Homer1) protein localisations in fixed mouse brain sections. However, the technique is readily adaptable to other types of targets and tissues.

## Highlights

• SMLM reveals contiguous scatters of GLT-1 molecules near excitatory synapses.
• Bassoon and Homer1 molecules are on average 120 nm apart.
• GLT-1 molecules occur closer to Homer1 than to bassoon.
• Three-colour 3D dSTORM is adaptable to various tissues and targets.

## 1 Introduction

### 1.1 The nanoscopic nature of astrocytes and their involvement in synaptic transmission

Over the last decade, neuroglia, formerly considered a mere neuron-supportive cell group, have emerged as active partners of neurons in information acquisition, processing, integration and storage [reviewed in [1], [2], [3]. Astrocytes and astrocyte-like cells can be found throughout the nervous system. Protoplasmic astrocytes reside in the grey matter where they occupy non-overlapping territories [4], [5], [6]. These cells derive their name from their star-like appearance as they extend several primary processes from the soma, which in turn give off secondary branches. From these, a myriad of delicate, nanoscopic protrusions emanate which confer a sponge-like appearance to the cells [reviewed in [7], [8]. This complex morphology makes direct physiological probing of astrocytes inherently problematic. The soma and stem processes make up the astrocyte’s microstructure visible under diffraction-limited optical microscopes. However, they only constitute 15% of the astrocytic cellular volume and about 20% of the cell’s surface area [4]. The bulk of an astrocyte is thus made up by the nanoscopic protrusions which mediate key astrocytic functions. In addition to their involvement in neuronal signal transduction, astrocytes play essential roles in brain homeostasis [9]. Astrocytes are part of the blood–brain-barrier [10] and operate the lymphatic system [11]. They also regulate local blood flow [12] and provide metabolic support through lactate shuttling [13], and glycogen synthesis and storage [14]. Astroglia are also involved in chemosensing and pH regulation [15], regulate overall energy balance and food intake [15], as well as sleep homeostasis [16]. Furthermore, these cells regulate neurogenesis, neuronal development and guidance [17] as well as synaptogenesis, and synaptic maintenance, elimination and plasticity [18], [19]. Astroglia also regulate water, ion and neurotransmitter homeostasis [20], [21], [22], [23], [24], [25]. Owing to their critical role in normal brain function and health, astroglia dysfunction can lead to, and is a hallmark of, most if not all neurodegenerative disorders; in the context of pathophysiology, astrocytes are a promising target for therapeutic intervention [26], [27], [28].

The capacity of astrocytes to co-ordinate signal transduction in the brain has been associated with their overall size: it is thought that the bigger the astrocyte relative to neurons, the more synapses it can spontaneously or simultaneously co-ordinate and possibly integrate [29], [30], [31]. Although neuronal activity remains the *sine qua non* of brain function, it is the capacity or ability of astrocytes to integrate them that might arguably affect the efficiency of brain function, and ultimately the apparent brain capacity [29], [32], [33].

The main signalling pathways by which astrocytes respond to neuronal activity at synapses are mediated by calcium [reviewed in [34], [35], [36]. Changes in synaptic activity are picked up by the receptors and transporters on the astrocytic membrane, 'interpreted' in the cytoplasm and often responded to through the release of calcium from intracellular stores leading to further downstream responses, such as release of signalling molecules that modulate neuronal activity [reviewed in [1], [37], [38].

The emerging realisation that astrocytes are fundamental components of nervous system function has prompted increased research efforts into astrocyte biology. However, due to the complex nano-anatomy of these cells, the molecular underpinnings of astroglia-neuron signalling have not been satisfactorily investigated or fully understood. One key methodological limitation in monitoring live astroglia is that conventional optical microscopes cannot resolve structures beyond the diffraction limit of ~250 nm in the imaging plane and ~600 nm in the z direction [39], [40] (see below). Therefore, electron microscopy (EM) has traditionally been used to study synapses as well as nearby astrocytic structures. With the aid of EM, the fine astrocytic processes can be seen projecting towards excitatory synapses [41], [42], enwrapping both the pre- and postsynaptic specialisations with a greater affinity for the postsynaptic elements [43], [44], [45]. These astrocytic protrusions, often called perisynaptic astrocytic processes (PAPs), show regional and developmental heterogeneity but can be found in close apposition to most excitatory synapses [reviewed in [7], [8], [46]. Because their diameter could be as small as 50 nm [8], [47], [48], they have traditionally only been visualised with 3D EM. However, 3D EM methods are costly, time-consuming and resource-demanding to operate. More importantly, even the most accurate immuno-labelling EM studies have not been able to provide a contiguous 3D picture conveying the spatial arrangement of key astroglial proteins and the underlying cellular structures on the nanoscale. Therefore, recent efforts to elucidate functional nano-organisation of astrocytes have concentrated on novel super-resolution microscopy techniques [49], [50], [51], [52], [53].

### 1.2 Breaking the diffraction limit

The physical nature of light as electromagnetic waves imposes a limit on optical resolution: in simple terms, the light source size cannot be smaller than the light half-wavelength or the main part of it. This diffraction limit determines the smallest resolvable distance, generally in accord with Ernst Abbe’s equation *d* = 0.5λ/NA (where λ is wavelength, and NA is the objective's numerical aperture), which in the case of conventional optical microscopes is normally 200 nm or higher. Thus, any point (infinitesimally small) source of light in a specimen will be represented as a 3D point spread function (PSF) characteristic of an optical system. The PSF thus provides a standard measure of resolution. Its shape determines the full width at half-maximum (FWHM) for a point-source intensity profile, a resolution criterion used extensively in image analysis.

Whilst some insights into fine astroglial morphology can be achieved by diffraction-insensitive imaging techniques employing conventional optics [45], [54], the ongoing revolution in microscopy has been providing new optical methods to increase resolution beyond the diffraction limit [reviewed in [39], [55], [56]]. The most widely used applications are structured illumination microscopy (SIM) [57], stimulated emission depletion (STED) microscopy [58] and single molecule localisation microscopy (SMLM) [59]. SIM is based on the concept of spatially varying excitation light intensity by using periodic fine patterns introduced into the excitation light path of a widefield microscope [60]. The technique has achieved a lateral resolution increase by a factor of 2 [61]. STED uses two laser beams; the first is a focused excitation laser that excites the fluorophores, and the second laser with doughnut-shaped intensity profile (or PSF) depletes the excitation of the fluorophores around the excitation spot. This systematically reduces the PSF size leading to increased resolution, up to 20 nm laterally and 30–40 nm axially [62]. SMLM focuses on algorithm-based determination of the precise location of a fluorescent molecule of a known size using PSF statistics. SMLM techniques include photoactivation localization microscopy (PALM) [63], fluorescent PALM (FPALM) [64], PALM with independently running acquisition (PALMIRA) [65] and stochastic optical reconstruction microscopy (STORM) [66]. The basic principle behind these techniques is the sequential and stochastic photoswitching of fluorophores or fluorescent proteins. Only a few fluorophores are switched on, imaged and localised per cycle, ensuring that the probability of having two or more overlapping PSFs is minimised. Consequently, the final super-resolved image is generated through the summation of thousands of acquired image frames.

Here, we describe a super-resolution microscopy approach to unravel the nanostructure of tripartite synapses. Our approach is based on the SMLM method direct STORM (dSTORM) which uses conventional fluorophore-labelled antibodies [67], [68], [69]. With this approach we are able to reconstruct the nanoscale localisation of individual astrocytic glutamate transporter (GLT-1) molecules surrounding presynaptic (bassoon) and postsynaptic (Homer1) protein localisations in fixed mouse brain sections.

## 2 Material and methods

Astrocytic nanoscopy with dSTORM entails tissue acquisition, preparation and processing as well as image acquisition, processing and analysis. The technique relies on tagging a molecule of interest with a fluorescent probe using antibodies. The fluorescence emission of the tag is then captured and its size and location are used as surrogates for the molecule of interest.

### 2.1 Optical set-up

Super-resolution images were recorded with a Vutara 350 commercial microscope (Bruker Corp., Billerica, US-MA) equipped with the SML biplane technology [70], [71]. The machine features two cameras: an Orca Flash 4.0 scientific complementary metal-oxide semiconductor (sCMOS) camera (Hamatasu) with frame rate at 50 Hz for capturing super-resolution images and a semiconductor charge-coupled devices (CCD) camera (Photometrics) used for standard widefield imaging. Images were recorded using a 60x-magnification, 1.2-NA water immersion objective (Olympus). Also used are excitation lasers: 647 nm (for Alexa 647), 561 nm (for CF568) and 488 nm (for Atto 488) and a 405 nm activation laser.

Commercial systems are also available from several other suppliers, for example NIKON and Zeiss, and many home-built systems including lower-cost options have been used for SMLM [72], [73]. Our configuration of the Bruker Vutara system does not offer changing the angle of illumination and uses standard epifluorescent illumination. However, changing the angle of illumination can increase contrast and hence many super-resolution imaging systems use total internal reflection (TIR) illumination (when imaging cells) or highly inclined and laminated optical sheet (HILO) illumination (when imaging thicker samples) [74]. In addition to the biplane method of 3D imaging other methods exist [74], for example astigmatism [75], double-helically arranged PSFs [76], [77] or interferometry [78]. Astigmatism is the most widely used method to achieve 3D capability. It offers a working range of 1 μm, which is similar to that achieved with biplane [70], [77]. On the other hand, double-helix approaches have a usable range of around 2 μm [77], potentially making it the method of choice for non-commercial set-ups.

### 2.2 Calibration and experimental PSF generation

For the analysis of the SMLM raw data it is helpful to generate an experimental PSF function instead of using a theoretical PSF to 1) fit the fluorophore localisations and 2) calibrate chromatic aberrations and align the individual channels. The experimental PSFs for the laser lines/fluorophores used can be generated by means of a TetraSpeck bead sample (Thermo, #T7279).

To prepare the calibration sample 1 µl TetraSpeck microspheres are sonicated for 10 min. 500 µl of water is added, the tube vortexed, sonicated again for 10 min and then vortexed again. Next, 100 µl poly-DL-lysine solution (1 mg/ml; Sigma, #P9011) is added to the centre of a No. 1.5 coverglass (for example: SLS #MIC3350; same quality and thickness as the coverslips used for imaging). After 10 min the lysine solution is aspirated and the coverslip air dried completely. 10–30 µl of the prepared 1:500 bead sample are pipetted onto the centre of the dried lysine spot and let stand for 10 min, then aspirated and the sample air dried completely. Finally, 3–5 µl water or Zeiss Immersol W 2010 is pipetted onto a glass slide (e.g. Henso, #7107) and the coverslip is inverted on top. The coverslip is then sealed with nail polish. The bead sample can also be used unmounted with a circular stage adaptor (Thermo, #A7816) and can be stored in phosphate-buffered saline (PBS, Sigma, #4417) at 4 °C. The imaging should also take place in PBS. To generate the experimental PSFs, a z stack (fifty 100 nm steps) of the TetraSpeck sample is imaged and the resulting PSFs are calculated.

### 2.3 Tissue acquisition

Here, we are using tissue from naïve animals. However, the method is easily adaptable for transgenic animals etc. It is crucial that all animal procedures be carried out in accordance with institutional and local government guidelines, with diligent consideration of the animal’s welfare at each step throughout. Animal procedures were conducted as mandated by the European Commission Directive (86/609/EEC) and the United Kingdom Home Office (Scientific Procedures) Act (1986). The images shown here were taken from three P160 (postnatal day 160) male C57BL/6J mice (Charles River, UK). When imaging older animals one has to keep in mind that tissue autofluorescence increases with age [79], [80]. As with all imaging approaches, a good signal to noise ratio with as little unwanted fluorescence as possible is crucial for the quality of the resulting image (see below).

**Fig. 1:**
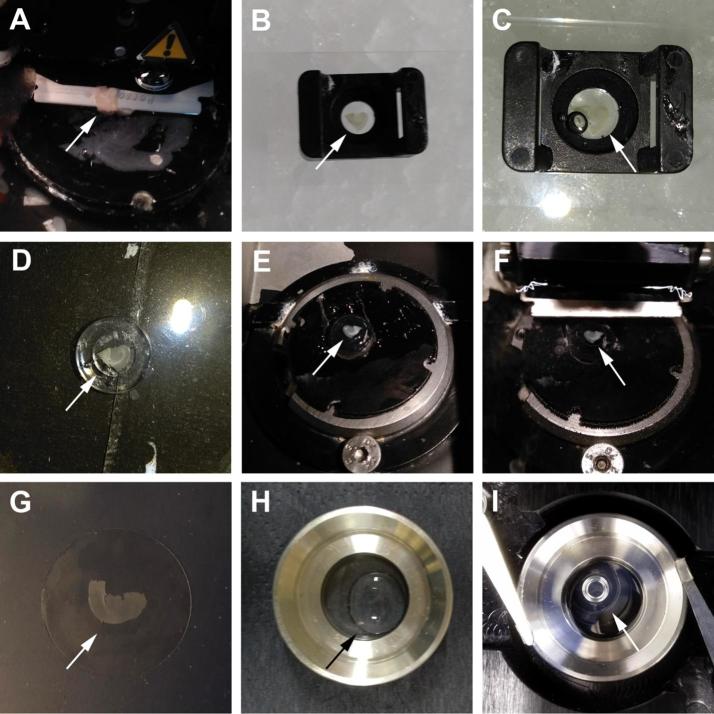
Tissue preparation for super-resolution imaging. (A) Acute, 350 μm brain sections are cut from a P160, male C57BL/6J mouse using a vibratome. (B) The sections are flattened out on a glass slide on ice. (C) 2% melted agarose is used to immobilise the tissue during reslicing. (D and E) The embedded tissue is glued to a vibratome stage. (F) The tissue is resliced into 30 μm sections. (G) For imaging, brain sections are placed on top of a coverslip. (H) Melted 2% agarose is used to immobilize the tissue. (I) The imaging chamber is filled with buffer, a coverslip is used to seal the chamber and the chamber is placed into the microscope for imaging. The arrows are pointing towards the tissue.

Perfusion was performed to cleanse the tissue from auto-fluorescing blood cells. Here, we describe the steps necessary for imaging of resliced ‘acute’ brain sections that can for be used for electrophysiological examination for instance. Nevertheless, the tissue can also be perfused with fixative directly and then sliced, stained and imaged. The animal was terminally anaesthetised with pentobarbital (i.p., 100 mg/kg; Merial, #R0270A) and laid supine. Testing the paw reflex ensured a stable and deep anaesthesia before continuing. Then, we exposed the pleural cavity, lifted away the sternum and freed the heart from surrounding connective tissue. We inserted a 26-gauge needle into the left ventricle, cut the right atrium and exsanguinated the animal with 10 ml PBS (stored at room temperature). Then, the brain was removed, the appropriate brain regions was dissected out and the brain was sectioned into 350 μm coronal sections using a vibratome (Leica, #VT1000S) (Fig. 1, A). These acute brain sections can be used for immediate live cell examination, or fixed and resliced for immunolabelling. For the fixation, the brain sections were incubated in pre-warmed (37 °C) 4% (w/v) paraformaldehyde (PFA, Sigma, #P6148, stored at 4 °C) in PBS for 30 min at 37 °C. Thereafter, the brain sections were washed three times for 20 min each with PBS. For reslicing, brain sections were flattened out on a glass slide on ice (Fig. 1, B). To immobilise the tissue during reslicing the brain sections were cast into 2% agarose (Lonza, #98200) in PBS (Fig. 1, C). We use small moulds that just fit around the brain sections. With the help of a needle, the brain/agarose block was removed from the mould (Fig. 1, D) and glued to the vibratome stage (Fig. 1, E). Care was taken to prevent any adhesive contacting the area to be sectioned. The tissue was then sectioned into 30 μm coronal sections (Fig. 1, F) and immediately used for immunohistochemistry or stored in PBS supplemented with 0.01% (w/v) sodium azide (NaN_3_; Sigma, #S2002) and 100 mM glycine (Sigma, #G8898) at 4 °C. The sodium azide was added to avoid contamination and the glycine was used to quench autofluorescence from residual fixatives.

When direct tissue labelling is desired, the mouse was first transcardially perfused with PBS as described above, followed by perfusion with 20 ml 4% PFA in PBS, noting the onset of fixation tremors. Good quality perfusion is necessary for good tissue preservation. After perfusion, the brain was removed and post-fixed overnight before being washed thrice with PBS to remove residual PFA. Fixed brains can be stored in PBS supplemented with 0.01% (w/v) sodium azide and 100 mM glycine at 4 °C for up to 3 months prior to sectioning.

The brain was then sectioned using a vibratome. First, we glued a block of 4% agarose in PBS to the vibratome stage and then glued the brain next to the agarose, to secure it during sectioning. Again, care was taken to avoid adhesive contacting the tissue. The brain was then sectioned into 30 μm coronal sections and immediately used for immunohistochemistry or stored in PBS supplemented with 0.01% (w/v) NaN_3_ and 100 mM glycine at 4 °C. We recommend using free-floating sections over cryosections in order to facilitate imaging in photoswitching buffer (see below) on inverted microscopes. The free floating sections can be mounted in stage adaptors and imaged in photoswitching buffer to achieve optimal blinking of the fluorophores for SMLM. Cryosections can be used but special isolators (e.g. Sigma, #S0810) have to be used to allow sealing of the tissue in buffer and inversion on top of the objective.

### 2.4 Tissue labelling

We used immunohistochemistry to label the astrocytic and neuronal proteins. All steps described here were performed gently rocking at room temperature (~21 °C) unless stated otherwise. Brain sections were briefly washed in PBS to remove any residual PFA. Then, they were incubated in 0.1% (w/v) NaBH_4_ (Sigma, #71320) in PBS (made fresh on the day) for 15 min to quench autofluorescence from residual free aldehydes [79], [81]. Glycine or NH_4_Cl can also be used for the quenching. Thereafter, sections were washed thrice for 5 min with PBS. As mentioned, tissue autofluorescence increases with ageing [79], [80]. In particular, the fluorescence from lipofuscin, a natural by-product of lysosomal activity in cells interferes with imaging as its emission spectrum overlaps with widely used fluorophores [79], [80]. To quench this tissue autofluorescence the sections were treated with 10 mM CuSO_4_ (Sigma, #C8027) in 50 mM NH_4_Cl (Sigma, #254134) (final pH = 5, stored at room temperature) for 10 min [82]. Another commonly used quencher is sudan black B. However, it introduces autofluorescence in the near infrared channel [83], [84] which interferes with the super-resolution imaging (see below). After quenching, the sections were washed quickly with deionised water, followed by three washes with PBS (5 min each).

Permeabilisation and blocking of the tissue was carried out using blocking buffer (PBS supplemented with 0.1% (w/v) saponin (PBS-S; Bio Basic, #SB4521) and 3% (w/v) bovine serum albumin (BSA; Sigma #A7906) for at least three hours. The blocking buffer can be made up in advance and stored at 4 °C for several days. Saponin is an amphipathic glycoside acting as a mild detergent. It reversibly permeabilises the cells by interacting with cholesterol and therefore has to be added throughout the staining process [85]. We recommend the use of glycosides such as saponin (at 0.1%) or digitonin (at 0.1%) when membrane proteins are to be imaged as the commonly used detergents triton X-100 (at 0.1–1%) and Tween 20 (at 0.2%) are non-selective and may remove proteins together with lipids [86]. To prevent unspecific binding of the antibodies we used BSA. Nonetheless, serum (recommended is serum from the animal in which the secondary antibody was raised), milk powder or other blocking agents can be used.

The brain sections were then treated with primary antibodies (Table 1) in PBS-S supplemented with reduced concentration of blocking agent (1% (w/v) BSA) overnight at 4 °C. This incubation step can also be performed at room temperature for only a few hours. Samples were washed briefly with PBS-S (~1 min) and then with PBS-S thrice for 10 min to remove surplus primary antibody. Afterwards, the sections were incubated with fluorescently-labelled secondary antibodies (Table 1) diluted in PBS-S for two hours. The best dye to use for SMLM is Alexa 647 (or Cy5) [87], [88]. It has excellent photoswitching characteristics and emits in the far-red spectrum, where tissue autofluorescence is comparatively low. For dual labelling Alexa 647 should be used in combination with CF568. Depending on the microscope system, Cy3B or Alexa 568 or Alexa 555 can be used instead. For triple labelling, Atto 488 should be used. Alexa 750 and DyLight 750 have also been used successfully [87], [89] but 750 nm lasers are not normally used in commercial microscopes. Different fluorophores display different blinking behaviour and the quality of reconstruction depends on the number of photons emitted in every photoswitching event. Commercially available antibodies are usually tagged with more than one fluorophore and not all fluorophores will ‘blink’ so that one cannot reliably estimate the number of actual molecules imaged. To avoid bleaching of the fluorophores, the sections have to be shielded from light (for example using aluminium foil to wrap the plate) from this point on. To avoid bleaching of overexpressed fluorescent proteins the brain sections should be kept covered in aluminium foil from the beginning. After incubation with the secondary antibodies, the sections were washed with PBS-S twice for 10 min and thrice with PBS for 5 min. Lastly, the sections were post-fixed using 4% PFA in PBS for 30 min to immobilize the antibodies in place to avoid movement and hence mislocalisations during imaging. This is followed by washing with PBS thrice for 10 min. Sections were stored covered in Scale U2 buffer [4 M urea (Sigma, #U6504), 30% (v/v) glycerol (Fisher, #BP229-1) and 0.1% (v/v) Triton X-100 (Sigma, #T9284)] [90] at 4 °C. This buffer clears the tissue to minimise autofluorescence. Scale U2 buffer can be prepared in advance and stored at 4 °C.

**Table 1:** Antibodies used. Ig = Immunoglobulin, RRID = Research Resource Identifier.

Primary antibodies used						
Antigen	Clone	Host	Supplier	Product code	RRID	Dilution
Bassoon	SAP7F407	Mouse	Novus	NB120-13249	AB_788125	1:250
GLT-1	Polyclonal	Guinea Pig	Merck	AB1783	AB_90949	1:500
Homer1	Polyclonal	Rabbit	Synaptic systems	160 003	AB_887730	1:250

Secondary antibodies used						
Antigen	Feature	Host	Supplier	Product code	RRID	Dilution
Guinea pig IgG	Alexa 647-conjugated	Donkey	Jackson	706–606-148	AB_2340477	1:500
Mouse IgG	CF568-conjugated	Donkey	Biotium	20,105	AB_10557030	1:250
Rabbit IgG	Atto 488-conjugated	Donkey	Sigma	36,098	AB_1137643	1:250

One has to keep in mind that the protocol described here uses complexes of primary and secondary antibodies, and the detected signals represent the position of the fluorophores rather than of the labelled proteins. Strategies exist to reduce this localisation precision error such as tagging primary antibodies directly with fluorophores, or using smaller antibody fragments such as nanobodies [91], aptamers [92], monomeric streptavidin [93] or the pore-forming bacterial toxin streptolysin O [94], [95]. Furthermore, the protein of interest can be modified with much smaller tags, using *hexa*-histidine [96], [97] or click chemistry approaches [98], [99], [100].

### 2.5 Imaging protocol

Just prior to imaging, the tissue is set atop a No. 1.5 coverslip (25 mm in diameter, SLS #MIC3350) and let to dry slightly (Fig. 1, G). Then, the coverslip is inserted into a 25 mm circular stage adaptor. Melted 2% agarose is pipetted on top of the tissue to immobilize the brain sections on the coverslip (Fig. 1, H). Then, the imaging chamber is filled with switching buffer (~1 ml). The buffer can penetrate the porous agarose gel and hence the tissue. Then a smaller coverslip (18 mm diameter) is set on top to seal the chamber (Fig. 1, I) which is then placed onto the microscope.

The switching buffer is needed to enable ‘blinking’ of the fluorescent dyes. It usually contains thiols such as mercaptoethylamine (MEA) or β-mercaptoethanol (β-ME) and oxygen scavenging enzymes such as glucose oxidase and catalase [66], [87], [101]. In multicolour experiments, different fluorophores might require slight changes to the imaging buffer [87], [88]. To increase the photoswitching efficiency chemicals such as cyclooctatetraene (COT) [102], [103] or Oxyfluor [104] can be added. Moreover, imaging can also be performed in mounting media such as Vectashield [103]. Here, we use a buffer protocol [105] containing 100 mM cysteamine and oxygen scavengers (glucose oxidase and catalase) as well as tris (2-carboxyethyl) phosphine (TCEP), which has been shown to reversibly quench cyanine dyes for improved photoswitching [106] (Table 2). The increased viscosity of the buffer due to the addition of glycerol leads to diminished interactions of the fluorophores and oxygen [107].

**Table 2:** Photoswitching buffer [85].

Enzyme Stock Solution (A)		
Ingredient	Concentration	Supplier and product number
10 µl catalase	20 µg/ml	Sigma, #C40
20 µl 1 M TCEP	4 mM	Sigma, #C4706
2.5 ml glycerol	50%	Fisher, #BP229-1
125 µl 1 M KCl	25 mM	Sigma, #P9333
100 µl 1 M Tris- HCl pH 7.5	20 mM	Sigma, #33742
5 mg glucose oxidase	1 mg/ml	Sigma, #G2133
Top up to 5 ml with distilled water and dispense into 50 µl aliquots and store frozen at −20 °C (for up to one year).		

Glucose Stock Solution (B)		
Ingredient	Concentration	supplier and product number
4 g glucose	100 mg/ml	Sigma, #G8270
4 ml glycerol	10%	Fisher, #BP229-1
Top up to 40 ml with distilled water and dispense into 400 µl aliquots and store at −20 °C (for up to one year).		

Reducing Agent Stock Solution (C)		
Ingredient	Concentration	supplier and product number
113.6 mg MEA-HCl	1 M	Sigma, #M6500
Top up to 1 ml with distilled water and store at 4 °C on the day of imaging. This solution can also be prepared in advance and stored at −20 °C for up to one year (do not refreeze).		

Just prior to imaging the above solutions are mixed:

50 µl Solution A, 400 µl Solution B, 100 µl Solution C, 450 µl PBS.

As a first step, a region of interest is selected by scanning the sample in standard epifluorescence mode using the CCD camera. Then, the sCMOS camera is used to confirm the region for super-resolution imaging. Now, the laser power is greatly increased to 0.6–6 mW/µm^2^ to induce photoswitching of the fluorophores. Sparse switching of fluorophores is desired to avoid overlapping PSFs. An image series of several thousand (usually 5,000–20,000) frames is recorded until most of the fluorophores have been documented and blinking is diminished. To maximise signal-to-noise ratio and to not split the blinking over multiple frames the exposure/recording time should on average match the time a single fluorophore emits photons - usually between 10 and 30 ms [108], [109]. To avoid bleaching and activating the other fluorophores recording should start in the red range of the spectrum and end in the blue range.

### 2.6 Image analysis

As mentioned, the acquired raw data consists of thousands of frames and up to millions of PSFs. During the analysis, the 3D position of every emitted organic dye is determined as accurately as possible. Most localisation algorithms fit a two- or three-dimensional Gaussian distribution at the centre of every detected fluorophore position and then sample the surrounding pixels [110], [111]. Here, we used the Vutara SRX software (version SRX 6.02.05). However, many freely available software packages, e.g. QuickPALM [112] or ThunderSTORM [113] exist also.

We used an in-house analysis suite run in MATLAB (version 2019a; MathWorks, Natick, US-MA) to identify synapses as opposing (within 500 nm) clusters of bassoon and Homer1. We also determined the nearest neighbour distances of GLT-1 molecules surrounding the identified synapses within a radius of 500 nm from the synapse center. The analysis suite is available for download (https://github.com/zhengkaiyu/LocSupRes). Moreover, we used Vutara SRX for cluster analysis of the bassoon and Homer1 localisations using DBScan and a minimum of 50 localisations per cluster and a maximum distance of 100 nm between localisations. We used GraphPad Prism (version 5.01; GraphPad Software, San Diego, US-CA) to plot the distances.

## 3 Results and discussion

Here, we describe a protocol for super-resolution imaging of tripartite synapses. We use the well described marker proteins piccolo for presynaptic structures, PSD95 for postsynaptic specialisations and GLT-1 as a marker of astrocytic membranes.

**Fig. 2:**
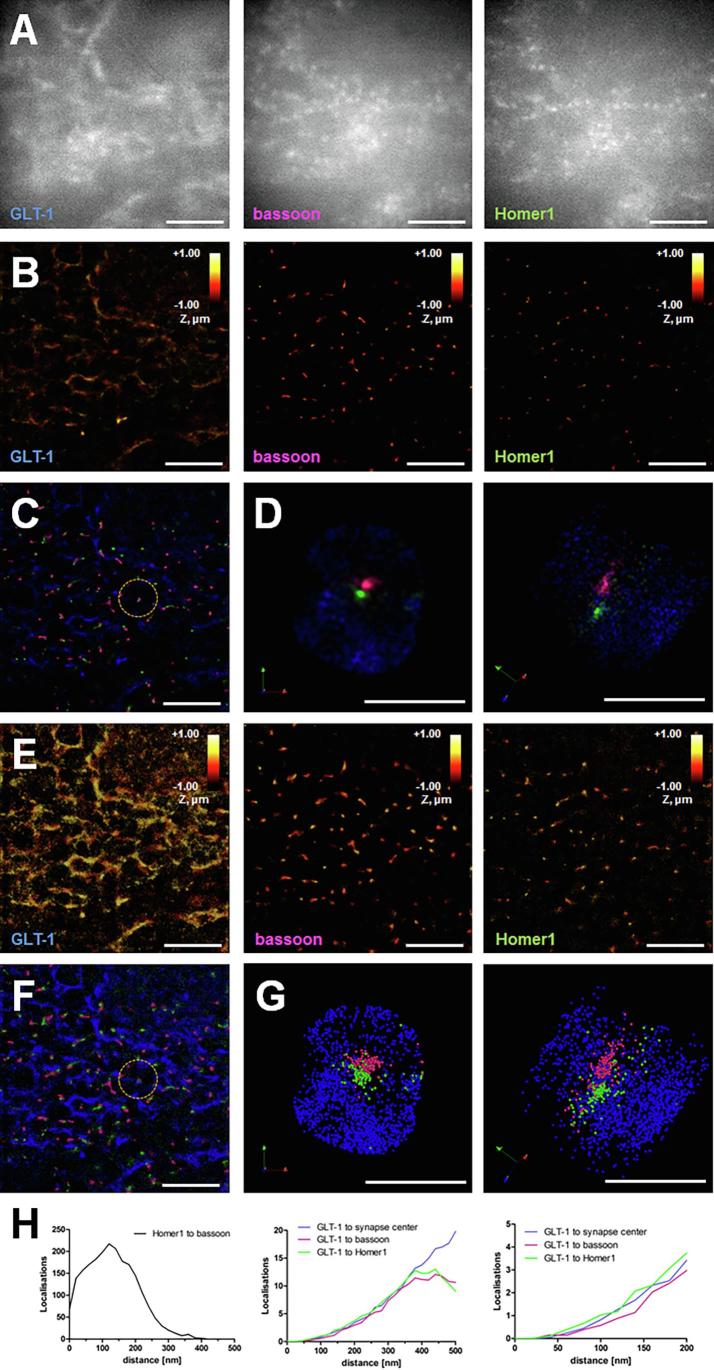
Super-resolution imaging of tripartite synapses in fixed mouse brain sections. (A) Wide-field images of astrocytic glutamate transporter GLT-1 (A1) and presynaptic protein bassoon (A2) and postsynaptic protein Homer1 (A3) in hippocampal CA1 area *stratum radiatum* in a fixed mouse 30 μm brain section (P160 male C57BL/6J). (B) 3D three-color dSTORM super-resolution imaging: Snapshots of molecular patterns for astrocytic GLT-1 (Alexa 647, B1), presynaptic bassoon (CF568, B2) and postsynaptic Homer1 (Atto 488, B3). The images are colour-coded according to the z position of the molecules in a 2 μm deep stack. (C) Merged image of images shown in B; GLT-1 (blue), bassoon (magenta) and Homer1 (green). Displayed are 2D projections of 3D SMLM molecular maps; label brightness reflects molecular density. (D) Close-up on the highlighted area in C; two viewing angles shown. (E) Same images as in B but as cloud representations of localised molecules with a constant diameter of 30 nm. (F) Merged image of images shown in E. (G) Close-up on the highlighted area in F; two viewing angles shown. (H) Nearest-neighbour distances (<500 nm) between synaptic molecules bassoon and Homer1 (H1). Distances of GLT-1 molecules to the center of identified synapses, bassoon or Homer1 molecules (H2). A zoomed in view of the distances up to 200 nm highlights that more GLT-1 molecules are closer to Homer1 than to bassoon or to the synapse center (H3). Scale bars = 5 μm (A-C and E-F) and 1 μm (D and G). (For interpretation of the references to color in this figure legend, the reader is referred to the web version of this article.)

After immunostaining, the tissue was prepared for super-resolution imaging and placed into the imaging chamber in photoswitching buffer. First, epifluorescence images of all three proteins were taken to identify the region of interest (Fig. 2, A). We imaged tripartite synapses in the CA1 *stratum radiatum* area of mouse hippocampus. The epifluorescence images show diffuse labelling with the GLT-1 antibody with some clustering throughout the neuropil, similar to what we and others have published before [50]. The synaptic molecules bassoon and Homer1 are scattered throughout the imaged tissue, forming clusters with roughly 1 µm distance between them [114]. After increasing the laser power, the ‘blinking’ of the fluorophores was registered and the final super-resolved images were generated (Fig. 2, B). The super-resolution images show the same area as in Fig. 2, A. Super-resolution microscopy revealed clusters of GLT-1, bassoon and Homer1 that were hardly identifiable in the epifluorescence images. Fig. 2, C is the merged image of the three super-resolved images in Fig. 2, B. Synapses are revealed with presynaptic (magenta) and postsynaptic (green) sites spaced apart from each other, revealing the synaptic cleft between them. The GLT-1 molecules (blue) form ‘clouds’ surrounding synapses with some clustering close to the synaptic molecules. The circled area in Fig. 2, C is depicted in two rotated views in Fig. 2, D. Presynaptic bassoon molecules are opposing postsynaptic Homer1 molecules. Surrounding the synapse are ‘clouds’ of GLT-1 molecules. Fig. 2, E–G represent the same images as in Fig. 2, B–D but the localisations are visualised as point clouds with a constant diameter of 30 nm. We measured the nearest neighbour distances of synaptic localisations in the area imaged (Fig. 2, H). We chose 500 nm as the cut-off point as it roughly represents the halfway point between two neighbouring synapses. The obersavation frequency for distances between bassoon and Homer1 localisations peaks at 120 nm (Fig. 2, H), a distance similar to published series [115]. The variety in distances might be due to activity states of the individual synapses in addition to mislocalisations and variation in labelling and imaging. GLT-1 molecules appear a little bit closer to the postsynaptic molecule Homer1 than they are to the presynaptic molecule bassoon or the synapse center (Fig. 2, H2 and H3).

**Fig. 3:**
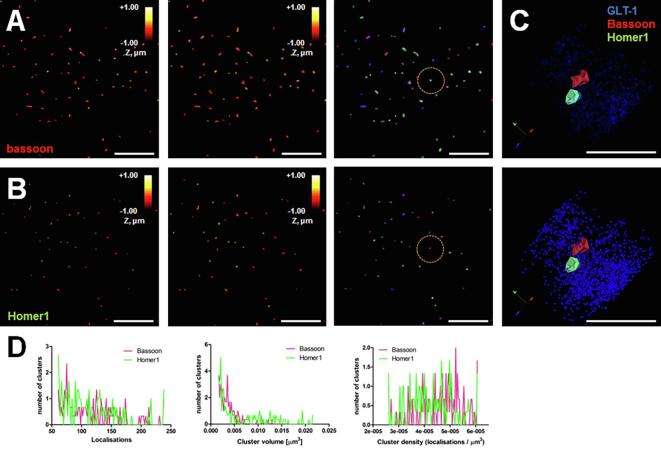
Cluster analysis of super-resolved synaptic molecules. (A) Clusters of super-resolved bassoon molecules in hippocampal CA1 area *stratum radiatum* in a fixed mouse 30 μm brain section (P160 male C57BL/6J) as in Fig. 2. The clusters are colour-coded according to the z position of the molecules in a 2 μm deep stack (A1 and A2) and visualised as cluster hulls as point splatted (A1), as point clouds (A2) and colour-coded as different as different clusters. **(B)** Clusters of super-resolved Homer1 molecules represented as in A. (C) Close-up of area highlighted in A and B. GLT-1 molecules (blue) surrounding the opposing synaptic molecules (bassoon in red and Homer1 in green) Merged image of images shown in B; GLT-1 (blue), bassoon (magenta) and Homer1 (green). Displayed are 2D projections of 3D SMLM molecular maps; label brightness reflects molecular density. (D) Cluster analysis on 84 synapses, depicting the number of localisations per cluster, the cluster volume and the density of localisations within the clusters. Scale bars = 5 μm (A and B) and 1 μm (C). (For interpretation of the references to color in this figure legend, the reader is referred to the web version of this article.)

We performed cluster analyses of the bassoon and Homer1 molecules across three imaged brain sections. We chose a minimum of 50 localisations per cluster and a maximum distance between two nearest neighbours of 100 nm as inclusion criteria. The analysis identified 84 synapses (distance between bassoon and Homer1 cluster center points less than 500 nm). Fig. 3 shows the identified clusters for bassoon (A) and Homer1 (B) represented as cluster hulls. Fig. 3, C is the same synapse highlighted in Fig. 2. Both, bassoon and Homer1, form clusters with varying numbers of localisations and resulting cluster volumes and densities, with small clusters being dominant (Fig. 3, D). As mentioned above, the number of localisations does not necessarily correspond to the actual number of molecules present. The different fluorophores behave differently in the buffer system, increasing the difficulty of multi-colour imaging.

## 4 Conclusion

The method described here details a super-resolution microscopy approach to image tripartite synapses on the nanoscale. The protocol is easily adapted to any three-colour imaging and can be used in a wide range of biological applications. Super-resolution imaging has allowed discoveries of the arrangement of cytoskeleton in axons, dendritic spine plasticity and molecular arrangements in pre- and postsynaptic specialisations [50], [116], [117]. Super-resolution imaging has increased our knowledge not only in fixed preparations but also in living brain slices [118] and living and freely behaving animals [119], [120], [121]. These techniques offer resolution far below that achieved with conventional microscopy techniques. Previously this was only possible with the help of 3D EM; a method that is costlier, technically demanding, time-consuming and does not allow imaging of living cells or animals. Here, we measured the distances between individual astrocytic and neuronal molecules. We found GLT-1 molecules located throughout the neuropil with some in close proximity to synapses as has been seen with conventional microscopy and EM [122]. Although morphological data cannot readily be obtained with SMLM it offers multi-colour imaging and easier 3D capabilities over a range of several microns. Glia cells have not seen a great deal of interest in the super-resolution field but with the advent of more convenient methodologies we will learn more about their functions in the healthy brain and what can go wrong in disease.

## 5 Equipment and supply list

### 5.1 Chemicals

**Table:** 

Material	Purpose	Provider
Antibodies	Immunochemistry	See Table 1
Agarose	Tissue immobilisation	Lonza, #98200
BSA	Blocking of unspecific antibody binding	Sigma #A7906
Catalase	Photoswitching buffer	Sigma, #C40
CuSO_4_	Quenching tissue autofluorescence	Sigma, #C8027
glucose	Photoswitching buffer	Sigma, #G8270
glucose oxidase	Photoswitching buffer	Sigma, #G2133
Glycerol	Tissue clearing, photoswitching buffer	Fisher, #BP229-1
Glycine	Quenching fixative autofluorescence	Sigma, #G8898
KCl	Photoswitching buffer	Sigma, #P9333
MEA-HCl	Photoswitching buffer	Sigma, #M6500
NaBH_4_	Quenching fixative autofluorescence	Sigma, #71320
NaN_3_	Tissue preservation	Sigma, #S2002
NH_4_Cl	Quenching fixative autofluorescence	Sigma, #254134
PBS	Buffer	Sigma, #4417
PFA	Fixation of brain and slices	Sigma, #P6148
Pentobarbital (Euthatal)	Anaesthesia	Merial, #R0270A
Poly-DL-lysine	Coverslip coating to improve adhesion	Sigma, #P9011
Saponin	Permeabilisation of cell membrane	Bio Basic, #SB4521
TCEP	Photoswitching buffer	Sigma, #C4706
Tris-HCl	Photoswitching buffer	Sigma, #33742
Triton X-100	Tissue clearing	Sigma, #T9284
Urea	Tissue clearing	Sigma, #U6504

### 5.2 Supply list

**Table:** 

Material	Purpose	Provider
Coverslips	Tissue immobilisation	SLS, #MIC3350
Glass slides	PSF calibration	Henzo, #7107
TetraSpeck beads	PSF calibration	Thermo, #T7279

### 5.3 Equipment

**Table:** 

Material	Purpose	Provider
60x, 1.2-NA objective	Imaging	Olympus
Circular stage adaptor	Holds coverslips for imaging	Thermo, #A7816
Lasers	Excitation and activation	Coherent
Orca Flash 4.0 sCMOS camera	super-resolution imaging	Hamatasu
CCD camera	Standard widefield imaging	Photometrics
Vibratome	Brain sectioning	Leica, #VT1000S
Vutara 350 microscope	Imaging	Bruker

### 5.4 Software

**Table:** 

Name	Purpose	Source
GraphPad Prism 5.01	Data analysis	GraphPad Software
MATLAB 2019a	Data analysis	MathWorks
Vutara SRX 6.02.05	Single molecule image acquisition and analysis	Bruker
